# Longitudinal Clinical and Minimally Invasive Readouts Enable Early Assessment of Disease Trajectory in Preclinical Infection Models

**DOI:** 10.3390/pathogens15090995

**Published:** 2026-09-20

**Authors:** Giorgio Montesi, Valentina Mocci, Fabio Fiorino, Donata Medaglini, Elena Pettini

**Affiliations:** 1 Laboratory of Molecular Microbiology and Biotechnology, Department of Medical Biotechnologies, University of Siena, 53100 Siena, Italy; giorgio.montesi@unisi.it (G.M.); valentina.mocci@student.unisi.it (V.M.); donata.medaglini@unisi.it (D.M.); 2 Department of Medicine and Surgery, LUM University Giuseppe Degennaro, 70010 Bari, Italy; fiorino@lum.it

**Keywords:** preclinical infection models, disease progression, longitudinal monitoring, minimally invasive readouts, *Salmonella* Typhimurium, machine learning, 3Rs

## Abstract

Murine infection models are essential for investigating host–pathogen interactions, disease progression, vaccine-induced immune responses and protection. Early identification of disease trajectories is important for both animal monitoring and high-quality biological sample collection. Although clinical and microbiological readouts such as body-weight changes, clinical scores, and circulating bacterial burden are routinely used to monitor disease progression, individual parameters often fail to capture short-term disease trajectory when analysed separately. Here, four longitudinal clinical and minimally invasive readouts (cumulative weight loss, day-to-day weight change, clinical score and blood bacterial load) were integrated using Machine Learning approaches to assess short-term disease trajectory and the occurrence of a fatal outcome within the subsequent three days. Two independently conducted *Salmonella enterica* serovar Typhimurium murine infection studies were analysed, with the first one used for model development and internal testing and the second one for independent external validation. Four classifiers (Random Forest, Support Vector Machine, XGBoost, and penalized logistic regression) alongside three ensemble strategies (majority voting, weighted voting, and stacked meta-learner) were evaluated in this experimental context. In the internal test set, all models achieved high performance, while in the independent external validation dataset, the stacked ensemble outperformed all other models achieving an accuracy of 0.96 and a specificity of 0.75. Although evaluated in a relatively small cohort using a single mouse genetic background and one *Salmonella enterica serovar* Typhimurium strain, these results indicate that the proposed Machine Learning framework, based on readily obtainable and minimally invasive longitudinal readouts, can serve as a valuable tool to anticipate short-term disease trajectory, support disease monitoring and improve experimental planning.

## 1. Introduction

Preclinical murine infection models remain essential for investigating host–pathogen interactions, elucidating mechanisms of disease progression, and testing potential prophylactic or therapeutic interventions. They provide a controlled experimental setup to capture the complexity of systemic infection and to identify quantitative markers of severity that can guide both mechanistic studies and welfare-oriented decision-making. Invasive infections caused by non-typhoidal *Salmonella* (iNTS) represent a major global health concern, particularly affecting young children, older adults, and immunocompromised individuals. For this reason, iNTS infections have been extensively studied using murine infection models, which have been essential in elucidating disease mechanisms and host responses [1,2,3,4]. In preclinical experimental settings, ensuring the protection of animal wellbeing in accordance with FELASA (Federation of European Laboratory Animal Science Associations) guidelines is a primary ethical requirement and the basis for evidence-based severity assessment. Early identification of disease worsening is therefore essential both for monitoring the transition toward critical disease stages and for supporting experimental planning and the appropriate definition of humane endpoints. Rigorous disease severity classification supports reproducibility, standardization, and the generation of biologically meaningful data from animal studies [5,6,7]. In this context, developing strategies capable of providing an early indication of worsening disease trajectory may contribute to more-efficient experimental designs by supporting the identification of informative sampling windows and endpoint planning. Such strategies align with the internationally recognized principles of the 3Rs (replacement, reduction, and refinement), which guide the ethical use of animals in research [8,9]. In particular, improved longitudinal monitoring can support more-informed decisions, facilitating the selection of informative sampling time points. This, moreover, may help in maximizing the biologically meaningful information obtained from each experimental cohort, and contribute to timely endpoint planning.

Routine monitoring in experimental infection models commonly relies on several clinical and microbiological readouts, including body-weight changes, clinical severity scores and circulating bacterial burden [7,10,11,12,13,14,15]. However, although individually informative, these parameters may not always provide a sufficiently clear prediction of short-term disease trajectory when considered in isolation [7,10,11,12,13,14,15]. Integrating information from these longitudinal measurements may therefore provide a more reliable representation of disease progression than reliance on individual readouts alone.

Machine Learning (ML) approaches are increasingly being adopted in biomedical research as valuable tools to capture complex, multivariate patterns associated with clinical outcomes. In human cohorts, ML approaches have been successfully applied to model mortality risk in large populations [16,17], to identify immune correlates of protection following vaccination [18,19], to predict adverse outcomes associated with infectious diseases [20] and to derive reproducible signatures for diagnostic and prognostic purposes [21]. Even though emerging evidence from veterinary clinical settings shows that ML models can aid decision-making across multiple indications, including risk prediction from routine clinical data, early detection of infectious diseases, and diagnostic case classification [22,23,24,25,26], the application of ML models to preclinical *in vivo* research, and particularly to experimental infection models in laboratory animals, remains at an early stage. Nevertheless, preclinical models represent a crucial intermediate step between mechanistic studies and translational applications generating longitudinal datasets that integrate multiple clinical and biological measurements and are therefore well-suited for ML analysis. However, these data are still often used mainly for descriptive or conventional statistical analyses; Therefore, expanding their use through ML approaches may help extract additional information from individual experiments, while supporting more-informed experimental planning, longitudinal disease monitoring and improved ethical management of laboratory animals.

In the present study, longitudinal clinical and minimally invasive readouts were integrated using different ML strategies in a murine model of intragastric infection with *Salmonella enterica* serovar Typhimurium strain D23580 (*S.* Typhimurium D23580), a well-established preclinical model of iNTS disease. In particular, cumulative weight loss, day-to-day weight change, clinical score and bacteraemia were evaluated in combination to assess whether their integration could provide an early indication of short-term disease trajectory and fatal outcome within the subsequent three days. The analytical strategy comprised four steps: (i) identifying informative patterns through exploratory analysis; (ii) training and testing multiple supervised algorithms in a first cohort of infected mice; (iii) integrating base models into three more ensemble approaches; and (iv) external validation of the finalized models in a second, independently conducted infection experiment.

## 2. Materials and Methods

### 2.1. Animal Infection Model

C57BL/6 mice were purchased from Charles River Italy (Lecco, Italy) and maintained under pathogen-free conditions in the animal facility of the Laboratory of Molecular Microbiology and Biotechnology (LA.M.M.B.), Department of Medical Biotechnologies at the University of Siena. The animals were housed in groups of up to six animals in cages (Tecniplast, Buguggiate, Italy) at a temperature of 20–24 °C, humidity rate of 55 ± 10%, with food and water *ad libitum*, under conditions complying with the 3R principles (replacement, refinement and reduction) according to the national guidelines (Italian D. Lgs n. 26 of 2014), in conformity with the European 2010/63/EU Directive on the protection of animals used for scientific purposes. Animal experiments were approved by the Italian Ministry of Health with authorization No. 371/2019-PR.

The invasive clinical isolate from Malawi *S.* Typhimurium D23580 [27] was grown in Luria–Bertani (LB) broth [tryptone (10 g/L, Oxoid, Basingstoke, UK), sodium chloride (10 g/L, Carlo Erba, Milan, Italy), and yeast extract (5 g/L, Oxoid, Basingstoke, UK)] to an optical density at 590 nm (OD590) of 0.35 and stored at −80 °C in 10% glycerol until use. Colony-forming units (CFU) were determined by plating serial dilutions onto LB agar plates followed by overnight incubation at 37 °C with 5% CO_2_.

Animals were then inoculated by the intragastric route with an infection dose of 10^8^ CFU of *S.* Typhimurium D23580 using a gavage needle (22 GA), as previously described [28], in a final volume of 100 μL/mouse of LB broth without any previous antibiotic pretreatment. The inoculum size was verified by plating appropriate dilutions onto LB agar plates. Upon infection, animals were monitored daily for body weight, clinical score, and survival. To minimize variability associated with the timing of longitudinal measurements, body-weight and clinical score assessments were performed at the same time each day. Blood bacterial burden was measured at defined time points by plating appropriate dilutions onto Salmonella–Shigella agar plates (Oxoid, Basingstoke, UK).

Both experiments (Study 1 and Study 2) were conducted as longitudinal survival studies, with predefined observation periods of 34 days for Study 1 and 27 days for Study 2. Animals were monitored throughout disease progression until spontaneous death, euthanasia upon reaching predefined humane endpoint criteria, or completion of the scheduled observation period. Completion of the observation period was not considered a fatal event. Animals reaching a clinical score of 5 were euthanized for animal-welfare reasons and not as part of a scheduled experimental sampling procedure. Euthanasia was performed by intraperitoneal administration of ketamine (Lobotor, 100 mg/kg; ACME S.r.l., Cavirago, Italy) and xylazine (Rompun, 8 mg/kg; Bayer S.p.A., Leverkusen, Germany).

### 2.2. Experimental Model and Data Collection

**Figure 1:**
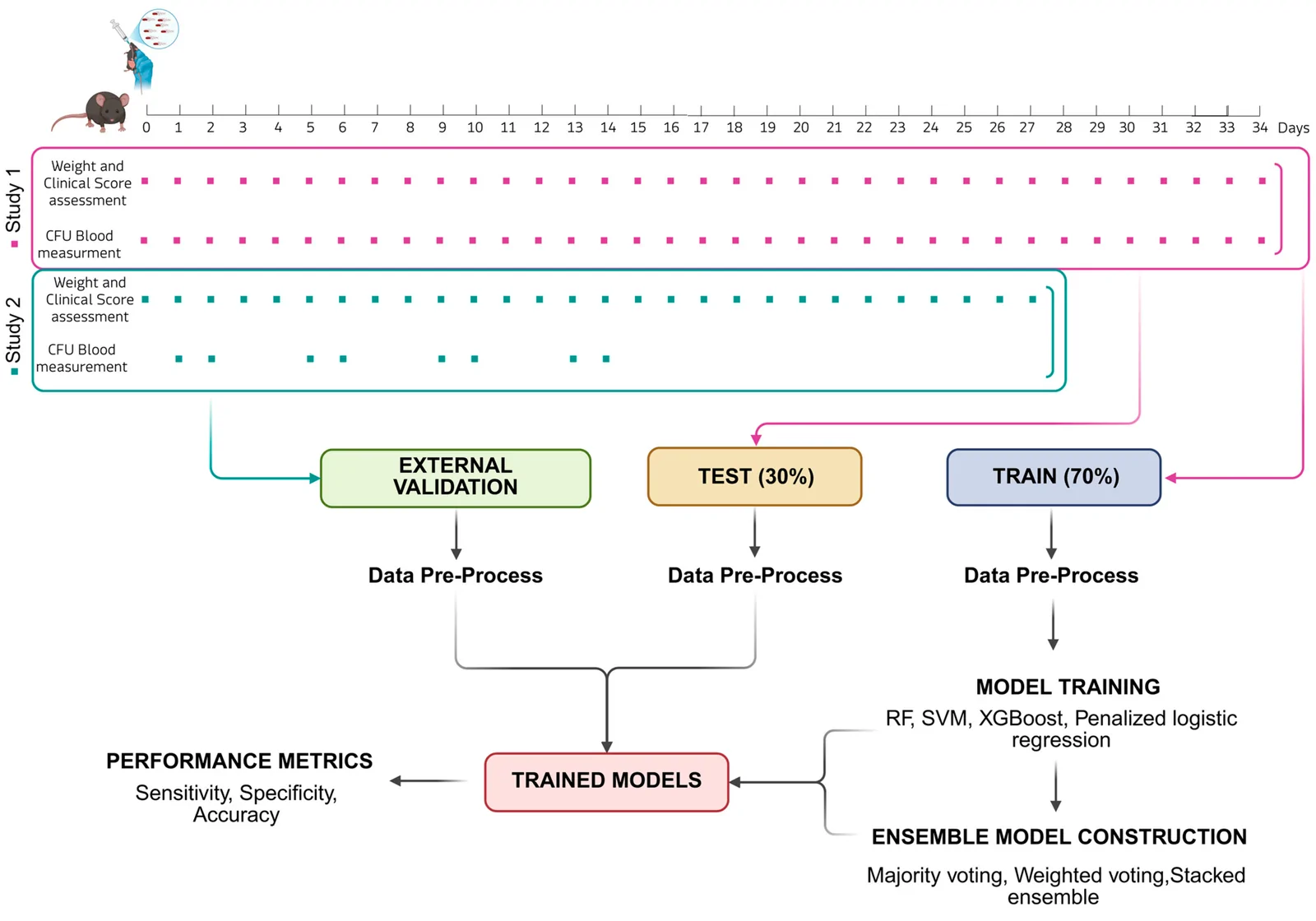
Experimental design and data analysis workflow. C57BL/6 mice, divided into two independent cohorts (Study 1 and Study 2) were intragastrically infected with *S*. Typhimurium. The predefined observation periods were 34 days for Study 1 and 27 days for Study 2. Completion of these periods was not considered a fatal event. Study 1 included 18 infected mice, of which 17 contributed eligible observations to the Machine Learning analysis, resulting in 82 observations used for model development and internal testing. Study 2 included 16 infected mice, of which 8 contributed eligible observations after application of the predefined inclusion criteria, resulting in 23 observations used exclusively for independent validation of the finalized models. Daily monitoring included weight and clinical score assessment in both cohorts. In Study 1, blood samples for CFU determination were collected on alternating days across cages to minimize stress and blood volume loss, while ensuring equivalent temporal coverage. Data from Study 1 (in violet) were used for model development (70% employed for ML model training, 30% used for internal ML model testing), whereas Study 2 (in green) served as an independent validation cohort. After preprocessing, four classifiers (Random Forest, RF; Support Vector Machine, SVM; XGBoost; and penalized logistic regression) were trained, and ensemble strategies (majority voting, weighted voting, stacked ensemble) were implemented. Model performance was evaluated in terms of accuracy, sensitivity, and specificity. Only observations with complete predictor data and sufficient subsequent follow-up to determine the three-day outcome were included in the ML analysis.

Two separate and independently conducted survival experiments, hereafter referred to as Study 1 and Study 2, were analysed (Figure 1). Study 1 was used for model construction, including training and internal testing, whereas Study 2 served as a separate experimental cohort reserved exclusively for external validation of the finalized models.

Study 1 consisted of three cages housing six animals each (n = 18), while Study 2 consisted of two cages housing eight animals each (n = 16). In both experiments, animals were monitored daily, and the following key physiological and clinical parameters were collected for each subject: (i) Cumulative weight loss, defined as the cumulative percentage weight loss from baseline. (ii) Daily weight variation, defined as the percentage change in weight relative to the previous day (day-to-day dynamics). (iii) Clinical score, defined as the measure used to assess the severity of disease in the infected animals, determined using a scale ranging from 0 (normal) to 5 (severe clinical condition requiring euthanasia according to the predefined humane endpoint criteria), based on the observation of different markers, such as fur aspect, behaviour, activity, posture and eyelids. Specifically, the severity of the disease was classified with: 0 absence of symptoms; 1, animal with piloerection and reduced motility; 2, animal with curved back and semi-closed eyes; 3, animal that takes more than 5 s to turn from supine position to prone position when placed on the back; 4, animal that cannot turn in a prone position from the supine position; 5, severe clinical condition requiring euthanasia according to the predefined humane endpoint criteria. (iv) Bacteraemia: circulating bacterial load expressed as CFU/mL of blood. Blood sampling was performed on alternating days across the cages, such that each animal was bled at longer intervals. This alternating design ensured equivalent temporal coverage across the cohort while adhering to guidelines aimed at minimizing blood volume loss and procedural stress.

For each experiment, observations were excluded if any of the four predictor variables (cumulative weight loss, daily weight change, clinical score, or blood CFU) were missing or if sufficient subsequent follow-up was not available to determine the three-day outcome, as detailed below. Eligibility was assessed at the observation level, and missing predictor values were not imputed, so that model development and evaluation were based exclusively on directly observed measurements with a clearly assignable three-day outcome.

For analytical purposes, a fatal outcome was defined as either spontaneous death or euthanasia required upon reaching the predefined humane endpoint. At each eligible observation time point, a binary outcome label, hereafter referred to as “fatal outcome within 3 days”, was assigned to indicate whether the animal experienced either spontaneous death or euthanasia upon reaching the predefined humane endpoint within the subsequent three days. The label was set to 1 when either event occurred within this interval and to 0 when the animal remained alive beyond the three-day window. To ensure complete outcome ascertainment, only observations for which the subsequent three-day period was fully covered by the experimental follow-up were considered eligible. Accordingly, observations from animals remaining alive at the end of the study were excluded when the corresponding three-day outcome window extended beyond the predefined observation period (day 34 for Study 1 and day 27 for Study 2).

### 2.3. Pre-Processing and Hyperparameter Tuning

The Study 1 dataset was split into training (70%) and test (30%) sets using stratification by outcome. The 70/30 split was selected as a commonly used hold-out strategy in supervised ML, retaining the majority of the available data for model development and hyperparameter tuning while preserving a separate subset for internal performance evaluation. All predictor variables were standardized using z-score transformation, with mean and standard deviation estimated exclusively from the training data within each cross-validation fold.

Four supervised learning algorithms were trained within an R 4.5.0 environment following tidy models v1.3.0 workflow: (i) Random Forest (RF; ranger v0.17.0), a tree-based model that aggregates the results of multiple decision trees to reduce overfitting; (ii) Support Vector Machine with radial basis function kernel (SVM; *kernlab v0.9-33*), a classifier that maps data into a higher-dimensional space to find the best boundary between classes; (iii) gradient boosting decision trees (XGBoost; *xgboost v1.7.11.1*), a tree-boosting algorithm that builds sequential models to correct the errors of the previous ones; (iv) penalized logistic regression with elastic net regularization (*glmnet v4.1-9*), a linear model used to predict binary outcomes, which includes regularization to avoid overfitting.

For each algorithm, hyperparameters controlling model complexity (i.e., number of variables sampled at each split and minimum node size for Random Forest; cost and RBF sigma for SVM; learning rate, tree depth, minimum node size, number of variables sampled at each split and loss reduction for XGBoost; penalty and mixing ratio for logistic regression) were tuned through a systematic grid search.

Model selection relied on stratified 5-fold cross-validation within the training set: data were partitioned into five equal folds, with four used for training and one for validation in rotation, ensuring balanced outcome proportions across folds. This procedure was repeated for all hyperparameter combinations (tune grid = 20), and average performance metrics were computed across folds. Optimization targeted multiple standard metrics including overall accuracy, sensitivity, defined as the ability to correctly classify true positives (i.e., mice with a fatal outcome), and specificity, defined as the ability to correctly identify true negatives (i.e., surviving mice), with specificity prioritized to minimize false-positive classifications of animals surviving beyond the subsequent three days.

Importantly, the test set (30% of the entire Study 1 dataset) was kept fully independent throughout model development and was used only for final performance evaluation, providing an unbiased estimate of generalization.

### 2.4. Ensemble Model Building

In addition to base models, three ensemble models combining predictions from the four base models were built: (i) a majority vote classifier, in which the final prediction in the class is predicted by at least three out of four models; (ii) a weighted vote, in which predictions are weighted by the specificity of each base model, giving more influence to those with higher specificity values; and (iii) a stacked ensemble, in which predictions from the base models are used as input for a second model (a penalized logistic regression), which learns how to optimally combine them. This meta-model was tuned to balance the contribution of each base model while preventing overfitting through regularization.

## 3. Results

In the present study, four longitudinal clinical and minimally invasive readouts (cumulative weight loss, daily weight variation, clinical score, and bacteraemia) were integrated using several ML approaches to assess short-term disease trajectory following *S*. Typhimurium infection. These parameters, selected for their established relevance in murine infection models, were evaluated across two independently conducted experimental studies.

### 3.1. Study Cohort

Two independently conducted infection studies, namely Study 1 and Study 2 respectively, were analysed (Figure 1 and Table 1). The first dataset (Study 1) was used for model construction, encompassing both training and internal testing phases. Out of 18 infected mice, 17 met the predefined inclusion criteria, yielding 82 longitudinal observations across the four clinical and minimally invasive readouts considered (cumulative weight loss, daily weight variation, clinical score, and bacteraemia).

The second dataset (Study 2) consisted of a separate experimental cohort and was reserved exclusively for external validation. It comprised 16 infected mice, of which 8 satisfied inclusion criteria, resulting in 23 eligible observations for model evaluation.

This design ensured a clear separation between the development and validation cohorts and allowed performance metrics to be assessed on an independent dataset.

### 3.2. Exploratory Data Analysis

To examine how cumulative weight loss, daily weight variation, clinical score, and bacteraemia related to short-term disease trajectory and fatal outcome, preliminary exploratory analyses were performed.

**Figure 2:**
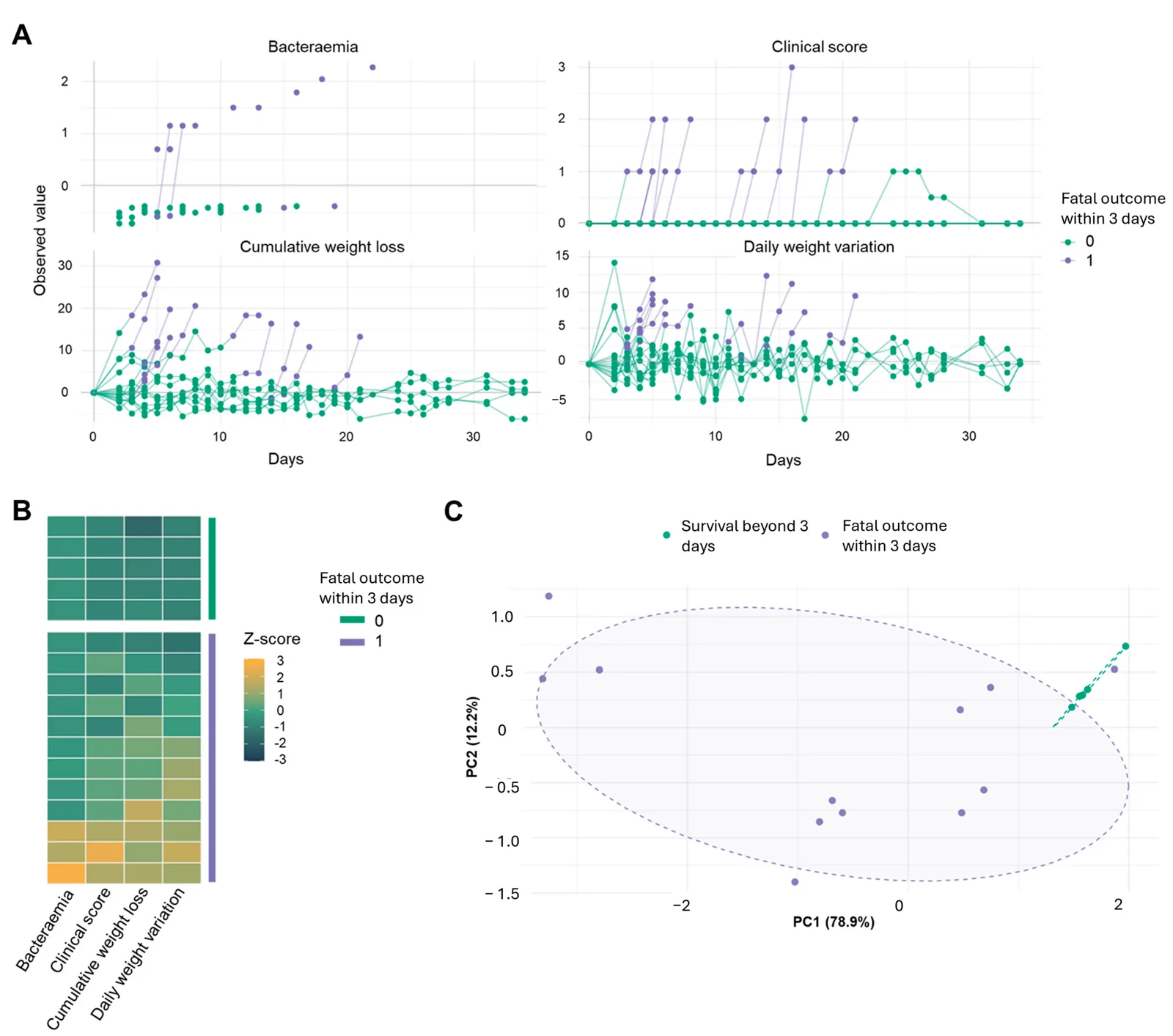
Exploratory analysis of clinical and minimally invasive readouts associated with short-term disease trajectory. (**A**) Longitudinal trajectories of the four measured variables—bacteraemia (CFU/mL, log scale), clinical score (ordinal 0–5 scale), cumulative weight loss (% from baseline), and daily weight variation (% change from previous day)—across the full observation period. Each dot represents a daily measurement from a single mouse. Observations preceding a fatal outcome within the subsequent three days are shown in purple, whereas observations followed by survival beyond this interval are shown in green. Time is indicated on the x-axis (days post-infection). (**B**) Heatmap of standardized variable values (z-scores) at the critical time point (i.e., last available observation preceding the fatal outcome or an equivalent matched time point for controls). Rows represent individual animals, and columns represent the four variables. Colour intensity reflects the degree of deviation from the variable mean: yellow tones indicate values above the cohort mean (worsening), while green tones indicate below-average values (more stable). Worsening patterns are apparent in observations preceding a fatal outcome. (**C**) Principal Component Analysis (PCA) of the same z-scored variables at the critical time point. The first two principal components are plotted, capturing 78.9% (PC1) and 12.2% (PC2) of the variance, respectively. Dots represent individual animals and dashed ellipses represent 95% confidence intervals; separation between observations preceding a fatal outcome within three days (purple) and those associated with survival beyond this interval (green) is visible along PC1, indicating multivariate structure among the predictors.

The time-course plot (Figure 2A) showed that animals experiencing a fatal outcome within the subsequent three days exhibited more pronounced cumulative weight loss, steeper day-to-day declines, a progressive worsening of the clinical score, and, when available, rising bacteraemia compared with animals surviving beyond this interval. However, none of these variables alone displayed a clear threshold capable of consistently distinguishing animals approaching a short-term fatal outcome.

For instance, regarding the clinical score (Figure 2A, top-right panel), some mice that progressed from a score of 0 to 1 subsequently experienced a fatal outcome within three days, whereas others showing a similar early trajectory recovered and returned toward baseline. Similarly, no clear cutoff was observed for either cumulative weight loss (Figure 2A, bottom-left panel) or daily weight variation (Figure 2A, bottom-right panel): some animals recovered despite losing more than 10% of their body weight, while others reached a fatal outcome despite smaller losses. A similar pattern was observed in the heatmap (Figure 2B), which summarizes the values of the four variables at the last available observation preceding the fatal outcome. Indeed, while a few mice displayed clear increases in all four variables shortly before death, for most animals experiencing a fatal outcome within the subsequent three days, such a sharp distinction was not consistently observable at the level of individual variables. In contrast, the Principal Component Analysis (PCA; Figure 2C), capable of capturing the linear joint effect of all four variables, revealed a clearer separation between observations associated with survival beyond three days and those preceding a fatal outcome.

Overall, animals nearing death exhibited a joint signature of increased cumulative weight loss, more pronounced day-to-day decline, higher clinical scores, and, where measured, elevated blood bacterial burden, whereas animals surviving beyond three days remained within milder, less coordinated fluctuations across the same variables. Interestingly, while individual variables alone were not always sufficient to distinguish observations preceding a short-term fatal outcome, their combined evaluation revealed a multivariate pattern associated with worsening disease trajectory. This observation prompted further investigation of the combined information provided by the four readouts through the application of various ML algorithms. 

### 3.3. Training on the Study 1 Dataset

To investigate the combined discriminatory information provided by the four variables considered (bacteraemia, clinical score, cumulative weight loss and daily weight variation), four ML classifiers, including Random Forest, SVM, XGBoost and penalized logistic regression, were implemented.

**Figure 3:**
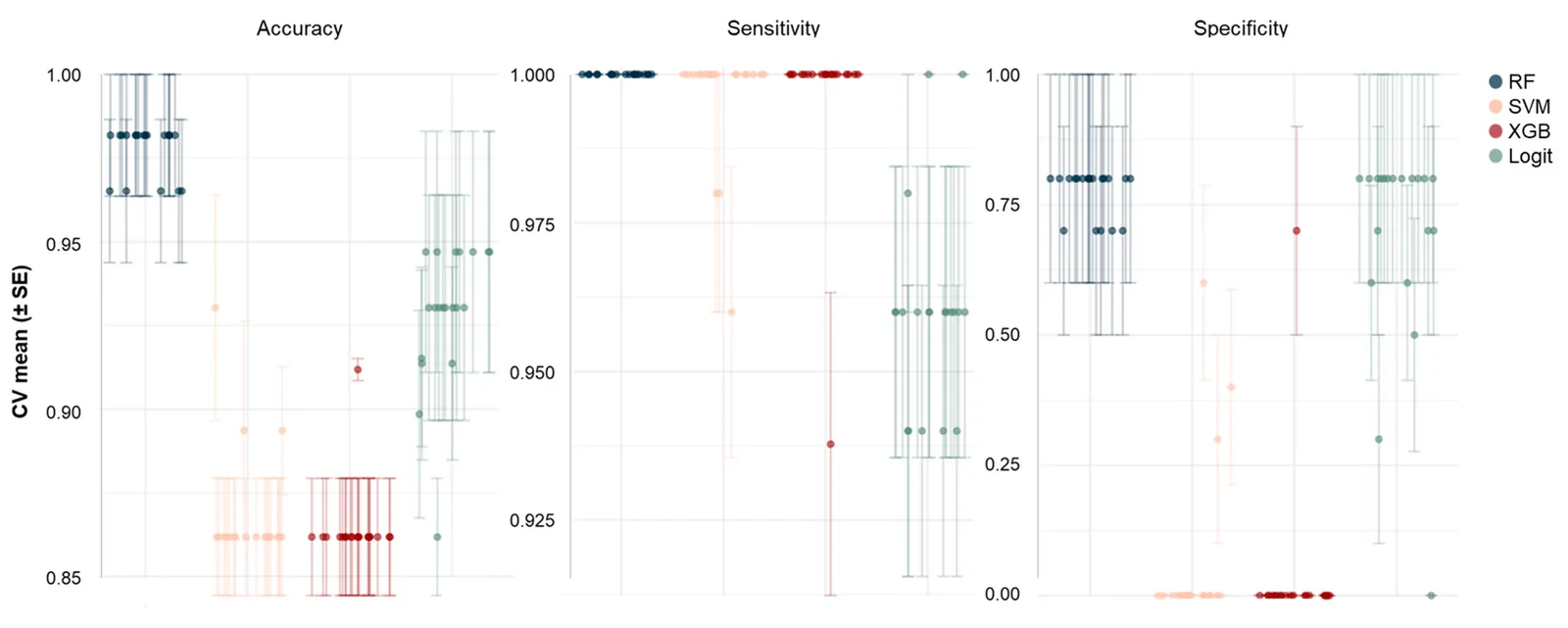
Cross-validated performance during hyperparameter tuning. Accuracy, sensitivity, and specificity values obtained during 5-fold cross-validation for each hyperparameter configuration tested across four classifiers: Random Forest (RF, dark blue), Support Vector Machine with RBF kernel (SVM, light orange), XGBoost (XGB, dark red), and penalized logistic regression (Logit, light green). Each dot represents the mean performance of a specific hyperparameter combination across folds, and the vertical bars indicate the associated standard error.

These four ML models were trained and tested on the Study 1 dataset. During training, all models underwent hyperparameter tuning through grid search combined with 5-fold cross-validation on the training data. This procedure systematically tested multiple parameter combinations to optimize predictive performance while minimizing overfitting, to finally select the best ones based on specificity, defined as the ability to correctly identify observations associated with survival beyond the subsequent three days. The cross-validated metrics obtained during this tuning phase were visualized (Figure 3): each point corresponded to a parameter configuration, with mean performance and associated variability shown across folds. All models achieved good performance in terms of accuracy (0.83–1) and sensitivity (0.9–1). However, specificity showed greater variability, particularly for SVM and XGBoost, which exhibited values ranging from 0 to 0.72 across different folds, suggesting reduced reliability in identifying true survivors under certain parameter settings.

The final set of hyperparameters selected as those yielding the highest specificity for each model (Supplementary Table S1) were then used to refit the models on the full Study 1 training dataset before evaluation on the independent test partition.

### 3.4. Independent Testing of Ensemble and ML Models on the Study 1 Testing Dataset

Based on the promising performance observed during training, all four individual ML classifiers were further evaluated on the independent test set of Study 1 using their respective optimized hyperparameters.

In addition to the base classifiers, three ensemble strategies, including a majority voting, a weighted voting (with weights defined as model-specificity values) and a stacked meta-learner ensemble model, were implemented to assess whether combining predictions could improve overall classification performance.

The meta-learner ensemble model selected a penalty of 0.144 and retained contributions from one SVM, one XGBoost, and 20 Random Forest models (Supplementary Figure S1). Models with higher stacking coefficients contributed more strongly to the final decision, while redundant or less informative members were effectively down–weighted.

All seven models (four base classifiers and three ensemble approaches) achieved high performance on the test set in terms of accuracy (ranging from 0.92 to 0.96) and sensitivity (1.00 for all models). However, specificity remained more variable across models, ranging from 0.50 to 0.75 (Table 2; Supplementary Figure S2). Notably, the stacked meta-learner and the SVM classifier reached the highest specificity (0.75), indicating better identification of observations associated with survival beyond three days.

### 3.5. External Independent Validation on the Study 2 Dataset

When tested on the external and independent Study 2 dataset (Figure 1), all models retained generally high sensitivity (0.89–1), indicating that most observations preceding a fatal outcome were correctly classified as positive. 

The stacked ensemble achieved the best overall performance, with a sensitivity of 1.00, specificity of 0.75, and an overall accuracy of 0.96 (Table 3; Supplementary Figure S2). This indicated its strong ability to correctly identify observations preceding a fatal outcome within the subsequent three days while reducing false-negative classifications. Observations from mice surviving beyond the three-day window were also generally identified as low risk, although the frequency of false-positive classifications varied across models.

Overall, these results indicate that the stacked ensemble model outperformed the others, maintaining a strong classification performance across both internal testing and independent validation datasets (Table 2 and Table 3; Supplementary Figure S2).

## 4. Discussion

In this study, four longitudinal clinical and minimally invasive readouts (cumulative weight loss, daily weight variation, clinical score, and bacteraemia) were integrated using multiple ML approaches to assess short-term disease trajectory in a murine model of *S.* Typhimurium infection. Their combined evaluation supported, through the stacked ensemble ML model, the identification of multivariate patterns associated with a fatal outcome within the subsequent three days.

Among the considered variables, cumulative weight loss remains one of the most widely adopted markers of disease severity, reflecting both metabolic alterations and the progression of systemic illness [10]. Its sensitivity makes it a valuable marker of health status, but reliance on rigid thresholds poses clear limitations. As highlighted by Brochut et al. [7], stringent cutoffs can lead to premature euthanasia of animals otherwise capable of recovery, while in other cases deterioration may occur before the threshold is reached. This limitation motivated our inclusion of a second, dynamic weight-based readout in the present analysis.

Unlike cumulative weight loss, which reflects the overall progression of illness and decreased wellbeing, day-to-day weight variation provides a more dynamic perspective, capturing short-term trajectories that may precede clinical collapse. In murine sepsis models, Osuchowski et al. [11] demonstrated that rapid and pronounced weight declines closely tracked the terminal phase of disease in non-survivors, highlighting the capacity of daily changes to capture short-term deterioration that might remain less evident when relying exclusively on cumulative measures.

Beyond weight-related parameters, clinical scoring systems offer a complementary, multidimensional view of animal wellbeing. By integrating non-invasive observations of fur condition, posture, mobility, behaviour, and responsiveness, they provide a structured evaluation of disease severity. Several studies have demonstrated their predictive value, showing that composite scores anticipate mortality with high accuracy in murine sepsis models and can serve as more-humane and reproducible endpoints than death itself [7,12,13]. In neonatal mice infection models, structured scoring has even outperformed conventional biomarkers such as circulating cytokines in predicting both survival and time to death [14]. Simplified 0–5 clinical scoring systems have also been shown to improve reproducibility in experimental settings [15]. In this study, a 0–5 scale from normal status (0) to severe clinical condition requiring euthanasia according to the predefined humane endpoint criteria (5) was applied, consistent with FELASA guidelines for standardized severity assessment, humane endpoint definition and the grimace scale [29,30].

Finally, blood bacterial load represents direct microbiological evidence of systemic infection and is strongly associated with outcome. Elevated bacterial counts in peripheral blood are consistently linked to increased mortality in murine models of invasive bacterial disease. In preclinical infection models, quantitative blood cultures have been correlated with fatal outcomes and heightened inflammatory responses in non-survivors [31,32,33]. These data confirm the role of bacterial dissemination as a marker of disease severity, providing a microbiological readout that complements host-based indicators such as weight metrics and clinical scores.

While these findings highlight the individual value of each parameter, the exploratory analyses also showed that no single readout consistently distinguished observations preceding a short-term fatal outcome. Rather, worsening disease was characterized by the joint behaviour of multiple clinical and microbiological measurements. ML approaches provide a flexible means of integrating such complementary variables in both linear and non-linear forms, allowing multivariate relationships to be evaluated without reliance on a single predefined threshold [34]. Building on this rationale, four supervised classifiers (RF, SVM, XGBoost, and penalized logistic regression) as well as three ensemble strategies (majority voting, weighted voting, and a stacked model) were implemented. Models were developed and internally evaluated on a discovery dataset (Study 1) and subsequently validated on an independent study (Study 2), maintaining good overall performance. This aspect provides further support for the use of an integrated approach based on these routinely collected readouts across separate experimental settings.

From an experimental perspective, early identification of animals approaching a fatal outcome within the subsequent three days could inform experimental decision-making, including the selection of biologically informative sampling windows and the scheduling of experimental procedures before disease reaches advanced stages. Integration of longitudinal clinical and minimally invasive readouts supports timely endpoint planning, contributing to the refinement of experimental procedures in accordance with the 3R principles. In this context, ML-based integration may further assist the earlier recognition of critical disease stages, helping align ethical considerations with scientific rigor.

Nonetheless, some limitations should be acknowledged. The model was developed and validated across two studies (Study 1 for model construction and Study 2 for external validation), representing a relatively limited sample size. Furthermore, analyses were restricted to a single host genetic background (C57BL/6) and one bacterial strain (*S*. Typhimurium D23580). Broader validation across different genetic backgrounds, infectious agents and doses, and experimental settings will be required to consolidate transferability of the proposed ML models. Accordingly, the trained models should not be assumed to transfer unchanged to other host–pathogen systems, where selection of disease-relevant predictors, model retraining and independent validation would be required. Looking ahead, incorporation of other immunological features, such as plasma cytokine profiles, may further enhance predictive performance and provide additional insight into host–pathogen dynamics.

In conclusion, the present work supports the feasibility of integrating routinely collected longitudinal clinical and minimally invasive readouts to obtain an early indication of short-term disease trajectory in a murine model of intragastric infection with *S*. Typhimurium. Cumulative weight loss, day-to-day weight variation, clinical score and bacteraemia provided complementary information that became more informative when evaluated jointly rather than in isolation. Among all tested models, the stacked ensemble meta-learner achieved the highest performance during both the model construction phase and validation on an external cohort. The analytical framework proposed should therefore be considered a valuable tool for integrating routinely available measurements to support longitudinal disease monitoring and the identification of informative experimental windows. By allowing early recognition of critical disease progression, this approach may support more-refined planning of experimental procedures and sampling and serve as a useful complement to predefined humane endpoint criteria. These elements contribute directly to improving adherence to the internationally recognized 3R principles, which promote ethically responsible animal research [8,9]. In particular, this work could contribute to the reduction principle by increasing the informational value obtained from each individual animal and potentially improving the efficiency of experimental designs. As highlighted by Richter and colleagues [35], innovation in study design is a key strategy to reduce animal use without compromising scientific rigor. In this perspective, integrative computational approaches may represent a valuable contribution to more-refined and ethically conscious experimental planning.

## Figures and Tables

**Table 1:** Study cohorts. The Study 1 dataset was employed for model development, while the Study 2 dataset was employed for external validation. * Observations = total number of time points contributing to the ML analysis.

Dataset	Total Number of Mice	Mice Included After Exclusion Criteria	Variables Observed	Total Observations (After Exclusion Criteria) *	Purpose
Study 1	18	17	4 (cumulative weight loss, daily weight variation, clinical score and bacteraemia)	272 (82)	Training + internal testing
Study 2	16	8	4 (cumulative weight loss, daily weight variation, clinical score and bacteraemia)	90 (23)	External validation

**Table 2:** Comparative performance of base and ensemble models on the test dataset. Comparative accuracy, sensitivity, and specificity for the four base classifiers, namely Random Forest, SVM, XGBoost, and penalized logistic regression (Logit), and the three ensemble strategies (majority vote, weighted vote, stacked). For each model, the three performance metrics are also shown as horizontal bar plots: fuchsia for accuracy, light purple for sensitivity, and dark purple for specificity.

	**Accuracy**	**Sensitivity**	**Specificity**	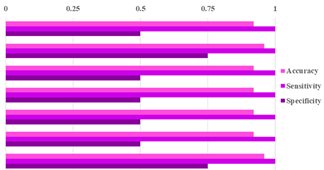
Random Forest	0.92	1	0.5	
SVM	0.96	1	0.75	
XGBoost	0.92	1	0.5	
Logit	0.92	1	0.5	
Majority vote	0.92	1	0.5	
Weighted vote	0.92	1	0.5	
Stacked	0.96	1	0.75	

**Table 3:** Comparative performance of base and ensemble models on the validation dataset. Comparative accuracy, sensitivity, and specificity for the four base classifiers, namely Random Forest, SVM, XGBoost, and penalized logistic regression (Logit), and the three ensemble strategies (majority vote, weighted vote, stacked). For each model, the three performance metrics are also shown as horizontal bar plots: fuchsia for accuracy, light purple for sensitivity, and dark purple for specificity.

	**Accuracy**	**Sensitivity**	**Specificity**	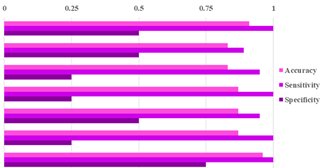
Random Forest	0.91	1	0.5	
SVM	0.83	0.89	0.5	
XGBoost	0.83	0.95	0.25	
Logit	0.87	1	0.25	
Majority vote	0.87	0.95	0.5	
Weighted vote	0.87	1	0.25	
Stacked	0.96	1	0.75	

## Data Availability

Data and code will be made available upon reasonable request to the corresponding author.

## Supplementary Materials

The following supporting information can be downloaded at: https://www.mdpi.com/article/10.3390/pathogens15090995/s1, Table S1: Tuned hyperparameters for each classifier. Final hyperparameter values selected after grid search and 5-fold cross-validation for each model. The table reports the computational engine, the hyperparameters tuned, the final selected values, and the total number of parameters optimized; Figure S1: Stacked model interpretation plots. Stacking coefficients assigned by the penalized logistic regression meta-model for the ensemble members. Higher coefficients indicate a stronger contribution to the final model. The model selected a penalty of 0.144, retaining one SVM (beige), one XGBoost (red), and 20 Random Forest models (blue). This configuration highlights the predominance of Random Forest members while preserving complementary signals from non-linear classifiers, ensuring both parsimony and balanced performance; Figure S2: Comparative performance of base and ensemble models on the test and validation datasets. Barplots report accuracy, sensitivity, and specificity for the four base classifiers, namely Random Forest (RF), Support Vector Machine with RBF kernel (SVM), XGBoost (XGB), and penalized logistic regression (Logit), and the three ensemble strategies: majority vote (Major.), weighted vote (Weight.) and stacked (Stacked). Performance is shown separately for the internal test set (light purple) and the external validation set (dark purple). Accuracy and sensitivity were consistently high across all models in both datasets, while specificity displayed greater variability. Notably, the stacked ensemble and SVM maintained the best balance between sensitivity and specificity, with the stacked model maintaining good overall performance across datasets.
