# Supplementary material for: Longitudinal Clinical and Minimally Invasive Readouts Enable Early Assessment of Disease Trajectory in Preclinical Infection Models
Source: Pathogens. 2026 Sep 20;15(9):995. doi: 10.3390/pathogens15090995 (PMC13610240; doi:10.3390/pathogens15090995)
Supplement: Supplementary file 1 [file pathogens-15-00995-s001.zip › Supplementary material_Montesi et al., 2026.pdf]

SUPPLEMENTARY MATERIAL

Supplementary Table S1. Tuned hyperparameters for each classifier. Final hyperparameter values selected after grid search and 5-fold cross-validation for each model. The table reports the computational engine, the hyperparameters tuned, the final selected values, and the total number of parameters optimized.

Model	Engine	Tuned hyperparameters	Final values	No. of tuned parameters
Random Forest	ranger	mtry, min_n, trees	mtry = 2; min_n = 28; trees = 1000	3
SVM	kernlab	cost, rbf_sigma	cost = 32; rbf_sigma = 0.0023	2
XGBoost	xgboost	mtry, min_n, tree_depth, learn_rate, loss_reduction, trees	mtry = 2; min_n = 2; tree_depth = 9; learn_rate = 0.0083; loss_reduction = 2.6e-08; trees = 1000	6
Penalized Logistic Regression (Elastic Net)	glmnet	penalty, mixture	penalty = 3.79e-09; mixture = 0.15	2

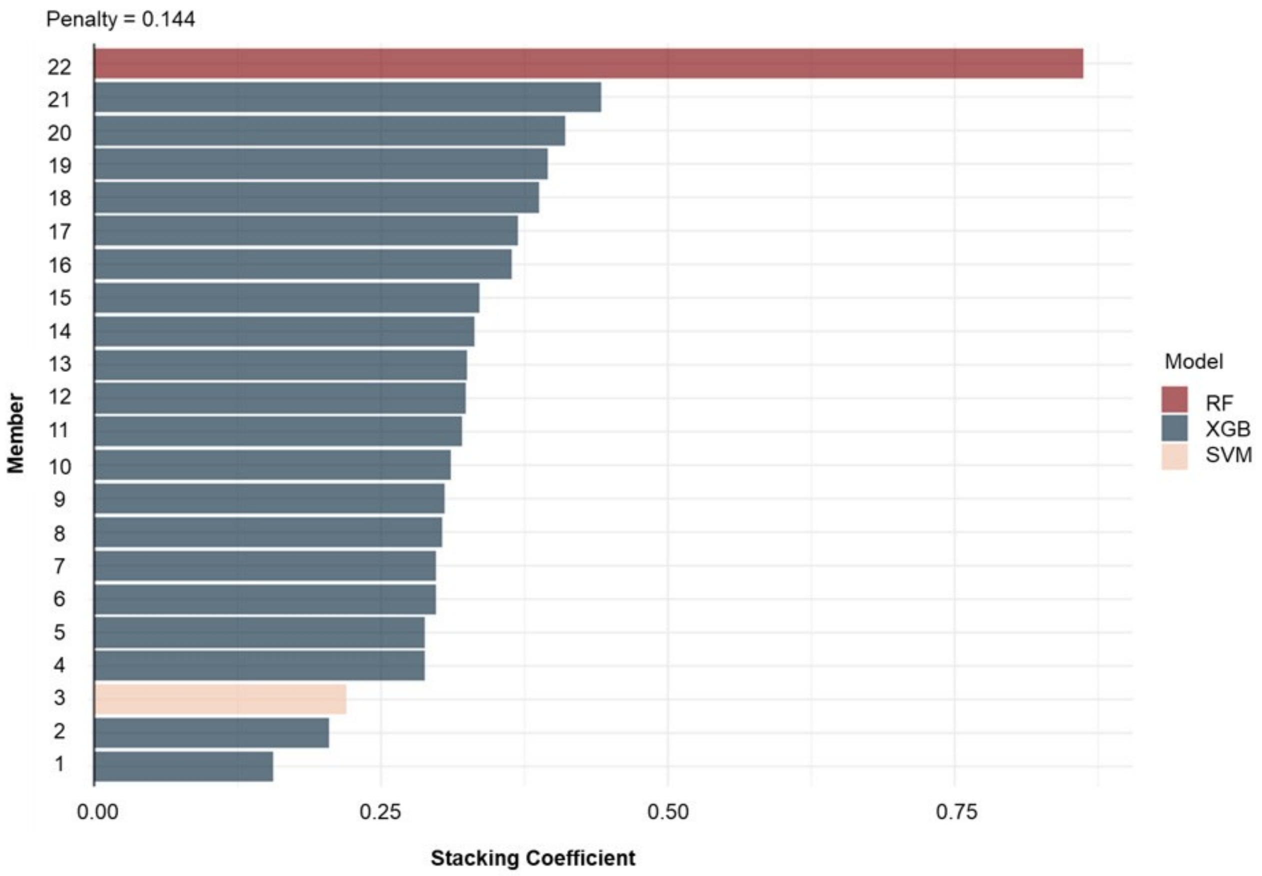

Supplementary Figure S1. Stacked model interpretation plots. Stacking coefficients assigned by the penalized logistic regression meta-model for the ensemble members. Higher coefficients indicate stronger contribution to the final model. The model selected a penalty of 0.144, retaining one SVM (beige), one XGBoost (red), and 20 Random Forest models (blue). This configuration highlights the predominance of Random Forest members while preserving complementary signals from non-linear classifiers, ensuring both parsimony and balanced performance.

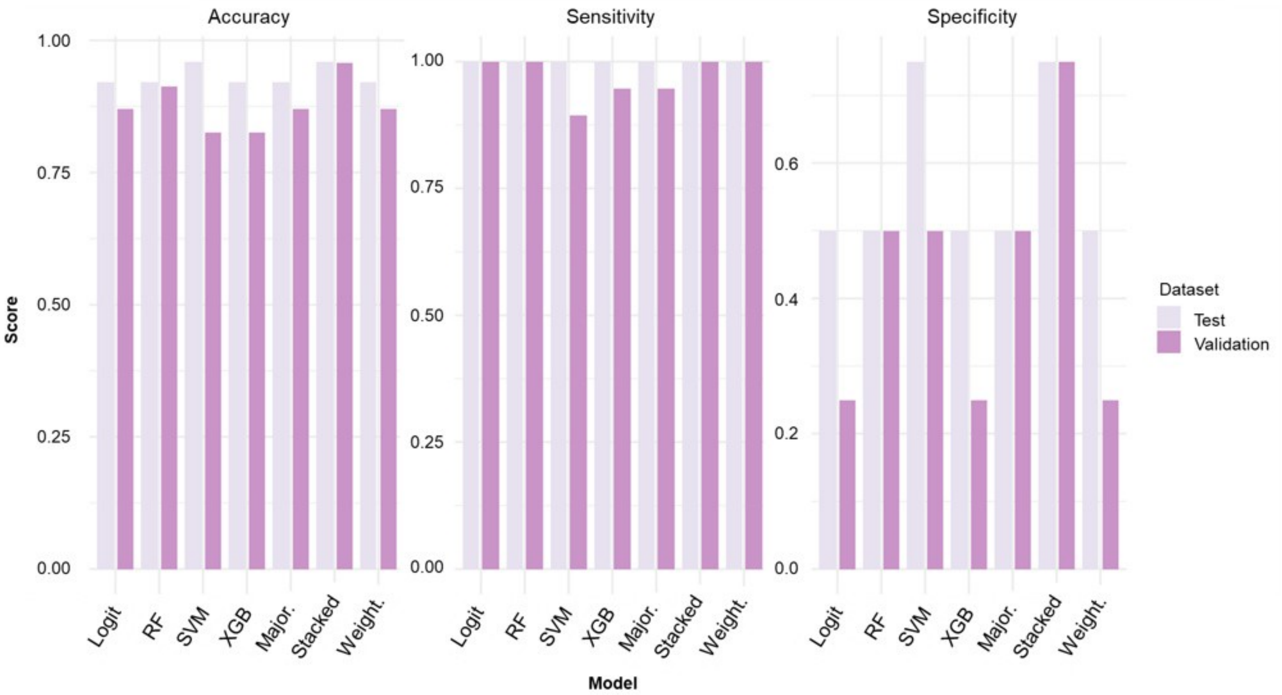

Supplementary Figure S2. Comparative performance of base and ensemble models on the test and validation datasets. Barplots report accuracy, sensitivity, and specificity for the four base classifiers, namely Random Forest (RF), Support Vector Machine with RBF kernel (SVM), XGBoost (XGB), and penalized logistic regression (Logit), and the three ensemble strategies: majority vote (Major.), weighted vote (Weight.) and stacked (Stacked). Performances are shown separately for the internal test set (light purple) and the external validation set (dark purple). Accuracy and sensitivity were consistently high across all models in both datasets, while specificity displayed greater variability. Notably, the stacked ensemble and SVM maintained the best balance between sensitivity and specificity, with the stacked model showing maintaining good overall performance across datasets.
